# Optimizing Cognitive Health: The Promise and Challenges of Physical-Cognitive Interventions for Dementia and Mild Cognitive Impairment

**DOI:** 10.31083/AP38762

**Published:** 2025-02-28

**Authors:** Yingchun Zeng, Guolong Zhang

**Affiliations:** ^1^Department of Nursing, Hangzhou Lin’an Traditional Chinese Medicine Hospital, Affiliated Hospital, Hangzhou City University, 311399 Hangzhou, Zhejiang, China; ^2^School of Medicine, Hangzhou City University, 310015 Hangzhou, Zhejiang, China; ^3^Respiratory Intervention Center, The First Affiliated Hospital of Guangzhou Medical University, 510120 Guangzhou, Guangdong, China

Cognitive function encompasses multiple domains, including language, perceptual-motor function, complex attention, executive function, learning and memory, and social cognition [1]. Cognitive impairment is defined as a decline in one or more of these domains, adversely affecting social function and quality of life [2]. Cognitive impairment and dementia are growing public health concerns, with estimates projecting nearly 150 million dementia cases globally by 2050, posing significant economic and social challenges [3]. Therefore, effective treatment methods and interventions for cognitive impairment are urgently needed to reduce this socioeconomic burden.

Currently, significant progress has been made in the risk identification, assessment, and early diagnosis of cognitive impairment. Research has also been conducted on drug treatments and intervention measures [4]. However, only a few drugs have been approved by the US Food and Drug Administration (FDA) for Alzheimer’s disease, showing limited improvements in cognition and function [5]. Considering the controversies surrounding pharmacological treatments, several nonpharmacological interventions have been developed to manage both behavioral and cognitive symptoms in patients with dementia or mild cognitive impairment. Common nonpharmacological interventions include cognitive training, cognitive rehabilitation, cognitive stimulation, exercise, peer support, psychoeducation, and care management. These interventions have also been recognized for their effectiveness by various health authorities [6].

These measures have demonstrated significant benefits for cognitive function. Recently, researchers have also begun exploring which types of treatments are most effective in managing cognitive impairment [1]. Network meta-analysis produces estimates of the relative effects between any pair of interventions in the network, and is widely applied to estimate for ranking the effectiveness of treatment interventions [7]. Venegas-Sanabria *et al*. [8] reported findings from a systematic review and Bayesian network meta-analysis investigating the effectiveness of various nonpharmacological interventions on global cognition in patients with mild cognitive impairment (MCI) and dementia [9]. One of the strengths of this analysis is that it includes a large number of studies and participants, covering a wide range of nonpharmacological interventions. This provides a holistic view of available treatment options and strengthens the generalizability of the results, which is crucial for understanding the relative efficacy of different approaches. The researchers systematically compared the effects of twelve nonpharmacological interventions on global cognition in cognitive impairment [9]. Overall, physical-cognitive rehabilitation was identified as the most effective intervention for unspecified cognitive impairment and dementia, while occupational therapy focusing on dual-task interventions was most effective for MCI. These findings underscore the importance of integrating cognitive and physical rehabilitation in managing cognitive impairment and can help develop specific treatment protocols for different stages of cognitive impairment.

An important finding of Venegas-Sanabria *et al*.’s study [8] is that combined physical exercise and cognitive interventions positively affected overall cognition and specific cognitive domains [7], consistent with recent research by Norouzi *et al*. [9] and Gallou-Guyot *et al*. [10]. Notably, the study highlights that combining physical and cognitive exercises can induce neuroplasticity and increase neurogenesis and synaptogenesis, thereby enhancing cognitive function [11]. Additionally, the combined intervention is a widely applicable strategy that shows positive effects on cognition for both healthy older adults and cognitively impaired populations, and it can be introduced either simultaneously or sequentially [9]. Therefore, combining physical and cognitive exercises can be used for both the prevention and intervention of cognitive impairment. As noted in previous studies, aging increases the prevalence of cognitive impairment [12]; thus, early identification of risk factors in older adults and implementing a combination strategy may be an effective pathway to prevent cognitive impairment.

However, the field must continue to explore the most effective physical-cognitive interventions for treating cognitive impairment. To our knowledge, few studies have focused on specific cognitive domains and long-term effects, with variability in study designs resulting in high heterogeneity, which limits the generalizability of these findings. Expanding outcome measures to include specific cognitive domains such as memory, executive function, and attention would provide a more comprehensive evaluation of intervention benefits and aid in developing more personalized cognitive intervention strategies [13]. A study design with longer follow-up periods, incorporating repeated measures of cognitive performance over time, is especially crucial for understanding the sustainability of cognitive benefits from the intervention. Additionally, developing and adhering to standardized protocols for nonpharmacological interventions could reduce variability and fully harness the potential of these interventions.
